# Assessment of metabolic and mitochondrial dynamics in CD4+ and CD8+ T cells in virologically suppressed HIV-positive individuals on combination antiretroviral therapy

**DOI:** 10.1371/journal.pone.0183931

**Published:** 2017-08-30

**Authors:** Jesse J. R. Masson, Andrew J. Murphy, Man K. S. Lee, Matias Ostrowski, Suzanne M. Crowe, Clovis S. Palmer

**Affiliations:** 1 James Cook University, Cairns, Australia; 2 Centre for Biomedical Research, Burnet Institute, Melbourne, Australia; 3 Baker IDI Heart & Diabetes Institute, Melbourne, Australia; 4 Instituto de Investigaciones Biome´dicas en Retrovirus y SIDA. Facultad de Medicina, Buenos Aires, Argentina; 5 Department of Infectious Diseases, Monash University, Melbourne, Australia; 6 Department of Microbiology and Immunology, University of Melbourne, Melbourne, Australia; University of Hawaii System, UNITED STATES

## Abstract

Metabolism plays a fundamental role in supporting the growth, proliferation and effector functions of T cells. We investigated the impact of HIV infection on key processes that regulate glucose uptake and mitochondrial biogenesis in subpopulations of CD4+ and CD8+ T cells from 18 virologically-suppressed HIV-positive individuals on combination antiretroviral therapy (cART; median CD4+ cell count: 728 cells/μl) and 13 HIV seronegative controls. Mitochondrial membrane potential (MMP) and reactive oxygen species (ROS) production were also analysed in total CD4+ and CD8+ T cells. Among HIV+/cART individuals, expression of glucose transporter (Glut1) and mitochondrial density were highest within central memory and naïve CD4+ T cells, and lowest among effector memory and transitional memory T cells, with similar trends in HIV-negative controls. Compared to HIV-negative controls, there was a trend towards higher percentage of circulating CD4+Glut1+ T cells in HIV+/cART participants. There were no significant differences in mitochondrial dynamics between subject groups. Glut1 expression was positively correlated with mitochondrial density and MMP in total CD4+ T cells, while MMP was also positively correlated with ROS production in both CD4+ and CD8+ T cells. Our study characterizes specific metabolic features of CD4+ and CD8+ T cells in HIV-negative and HIV+/cART individuals and will invite future studies to explore the immunometabolic consequences of HIV infection.

## Introduction

Metabolic dysfunction of immune cells in HIV-positive individuals is emerging as a hallmark of HIV infection, with important implications in disease pathogenesis and progression [1–4]. It is now recognized that glucose transporter 1 (Glut1), the major transporter of glucose on immune cells, is selectively essential for CD4+ T cell activation and effector function [5]. Previous work has shown that Glut1 is upregulated on CD4+ T cells in HIV-positive individuals irrespective of treatment status, and that this is associated with systemic immune activation. Furthermore, increased percentage of circulating CD4+Glut1+ T cells is inversely correlated with CD4+ T-cell count [6].

The expression and trafficking of glucose transporters to the T cell membrane allows glucose uptake by the cell, where it is broken down by glycolysis and oxidative phosphorylation (OXPHOS) [7–9]. In periods of high energy demand, Glut1 is up-regulated to fuel glycolytic metabolism, even in the presence of oxygen, to facilitate biomass production necessary for cell growth and proliferation [7,10,11]. There is often a simultaneous up-regulation of oxidative phosphorylation and high ATP production that coincides with an increase in mitochondrial biogenesis even when glycolysis predominates [7,12–15]. Since HIV infection is associated with changes in glucose metabolism in CD4+ T cells during HIV infection, the bioenergetics of the mitochondria may be called into play [6,13].

The shifts in metabolic profiles among T cell subpopulations vary depending on their activation and differentiation states. Quiescent inactivated T cells use either fatty acid oxidation (FAO) or glucose-derived pyruvate oxidation [16,17]. Upon stimulation, quiescent T cells differentiate into metabolically active T-effector and memory cells, which are regulated by PI3K/AKT/mTOR signalling to facilitate glucose uptake [9,18–20]. Despite the intimate link between nutrient metabolism, immune cell differentiation and function, the impact of HIV infection on mitochondrial dynamics is still largely unknown [21].

In this study, we analysed the metabolic phenotypes of T cells obtained from HIV uninfected individuals and virologically suppressed HIV-positive persons on cART. We examined Glut1 expression, mitochondrial density, mitochondrial membrane potential (MMP) and reactive oxidative species (ROS) production in total CD4+ and CD8+ T cells and their subpopulations to enhance our understanding of the bioenergetic changes in T cells during HIV infection.

## Materials and methods

### Study participants

The study population included HIV-positive patients on cART (HIV+/cART), and HIV seronegative controls (HIV-negative). Participating individuals were recruited from the community and the Infectious Diseases Unit at The Alfred Hospital in Melbourne, Australia. Approval for this study was obtained from the Alfred Health Human Research Ethics Committee, and informed consent was obtained from each participant. Blood samples from individuals recruited in Melbourne were collected in citrate anticoagulant tubes and processed within 1 hour of venepuncture to isolate and cryopreserve peripheral blood mononuclear cells (PBMCs). All participants with self-reported co-infection with hepatitis C virus (HCV), active malignancy, vaccination, physical trauma, or surgery within 3 weeks prior to participation were excluded from this study.

### Peripheral blood mononuclear cell preparation

Density gradient centrifugation (Lymphoprep, Axis Shield, Dundee, Scotland) was used to isolate PBMCs, as previously described [22], and cells were cryopreserved in 10% dimethyl sulfoxide (Sigma–Aldrich, St Louis, Missouri, USA) and 90% autologous plasma.

### Immunophenotyping

Cryopreserved PBMCs (>90% viability) were thawed in supplemented RPMI-1640 medium (10% human serum, penicillin/ streptomycin (Invitrogen), 2 mmol/l l-glutamine (Invitrogen, Carlsbad, California, USA)), before being stained on ice for 30 minutes as previously described [6]. Cells prepared for analysis of total CD4+ and CD8+ populations were stained with fluorochromatic antibodies provided by BD Bioscience using 2.5 μl of CD3 (PE), CD4 (PE-Cy7), and CD8 (APC-Cy7) antibodies. For analysis of CD4+ subpopulations, 2.5 μl of CD3 (APC; BD Bioscience), 2.5 μl of CD4 (PerCP-Cy5.5; BD Bioscience), 1 μl of CD45RA (APC-Cy7; BioLegend), 3 μl of CCR7 (PE-Cy7; BD Bioscience), and 1.5 μl of CD27 (PE; BD Bioscience) antibodies was used. Analysis of CD8+ T cell subpopulations used a similar staining strategy to the CD4+ T cell subpopulation method, with the substitution of 2.5 μl of CD3 (PerCP-Cy5.5; BD Bioscience) in place of the APC version and 2.5 μl of CD8 (APC; BD Bioscience) in place of the CD4 to gate the total CD8+ T cell population. All stained cells prepared were washed and resuspended in 300 μl of 1× PBS prior to flow analysis or prior to subsequent staining with mitochondrial dyes.

### Glucose transporter-1 detection

The Glut1 antibody [MAB1418 clone (R&D Systems, Minneapolis, Minnesota, USA)] conjugated with the FITC fluorophore (488 nm) (Filter: 530/30; Mirror: 502) was used to detect Glut1 on naive (N), central memory (CM), transitional memory (TM), effector memory (EM), and terminally differentiated (TD) CD4+ and CD8+ T cells, following the nomenclature described by Chomont et al. and Yap et al [23,24]. Cells were stained as described [6], acquired on a BD FACSCanto^TM^ II flow cytometer, and analysed using FlowJo software, version 8.8 (Tree Star Inc., Ashland, Oregon, USA).

### Mitochondrial dynamics

MitoTracker green (ThermoFisher Scientific), a fluorescent mitochondrial stain used to quantify mitochondrial density within individual cells, was used at a final concentration of 19 nM in pre antibody-stained cells resuspended in 100 μl of 1× PBS. Cells were incubated at 37 ˚C for 30 minutes before being washed once, and centrifuged at 1500 rpm for 10 minutes using a Beckman Coulter^TM^ GS6 centrifuge (Serial number: GB93B07). Supernatant was removed and the pellet resuspended in 1× PBS. MitoTracker green signal was measured using flow cytometry and was detected within the FL1/FITC channel.

The mitochondrial membrane potential (MMP) was also assessed in HIV-negative and HIV+/cART participants. The MMP is a major component of the proton motive force which increases during ATP synthesis and decreases when exposed to apoptotic stimuli [25]. MMP was measured using 3,3′-dihexyloxacarbocyanine iodide (Dioc_6;_ ThermoFisher Scientific), a fluorescent lipophilic dye which accumulates in mitochondria due to their inherently large negative membrane potential and measures reduction of mitochondrial membrane integrity reflecting pro-apoptotic signalling. It was used at a final concentration of 20 nM in pre antibody-stained cells resuspended in 100 μl 1× PBS. The cell solution was incubated, washed and resuspended as previously mentioned. Dioc_6_ signal was also analysed within the FL1/FITC channel.

MMP was also measured using MitoTracker red (ThermoFisher Scientific), a fluorescent dye that stains mitochondria in live cells, with its accumulation being dependent on MMP. This was used at a final concentration of 5 nM in pre antibody-stained cells resuspended in 100 μl 1× PBS. MitoTracker red signal was measured using flow cytometry and was detected within the APC channel (640 nm) (Filter: 660/20; No Mirror).

ROS production was measured using Hydroethidium (HE; ThermoFischer Scientific), a fluorogenic probe that detects intracellular superoxide free radicals, at a final concentration of 2.0 μM in 100 μl pre antibody-stained cells resuspended in 100 μl 1x PBS. ROS MFI was measured using flow cytometry to detect the PerCP-Cy5.5 fluorophore (488 nm) (Filter: 670; Mirror: 655).

### Statistical analysis

Using GraphPad Prism (version 6.0; GraphPad Software, La Jolla, California, USA) or SPSS (version 23; IBM statistical software, Armock, New York, USA) nonparametric data was analysed with Mann–Whitney testing for comparison of unpaired data, while Wilcoxon signed-rank test was used when comparing paired data. Pearson correlation coefficient was used to analyse parametric correlations while spearman rank correlation coefficient was used to analyse non-parametric correlations. P values < 0.05 were considered significant.

## Results

### Participant characteristics

A total of 31 participants, 18 HIV-positive individuals on cART and 13 HIV-negative control individuals, were recruited (Table 1). As expected the percentage of CD3+CD4+ T cells was significantly lower in HIV+/cART when compared to HIV-negative individuals (P<0.0001); absolute CD4+ T cell counts were not evaluated in HIV-negative individuals. The ratio of CD4+ and CD8+ T cells also differed significantly (P<0.0001).

**Table 1:** Clinical characteristics of study population.

Variables	HIV+/cART (25^th^– 75^th^ Quartile)	HIV-negative (25^th^– 75^th^ Quartile)	P-value
N	18	13	-
Sex (Male/Female)	16 / 2	11 / 2	-
Age (Median) years	52 (39.5–57.0)	45 (36.5–51.0)	0.09
CD4+ T cell count (Median) cells/μl	728 (616–1174)	-	-
Time on cART (Median) years/SD	15.2 (6.0–20.4)	-	-
Median % CD3+CD4+ T cells	39 (35–48)	51 (48–69)	<0.0001
Median CD4+/CD8+ T cell ratio	0.8 (0.6–1.1)	1.6 (1.3–2.4)	<0.0001
Viral load (Median) copies/ml	<20	-	-

cART: combination antiretroviral therapy. Continuous variables are expressed as median (percentile). The nonparametric Mann-Whitney U test was used to evaluate differences between subject groups.

### The frequencies of Glut1+ cells are highest among naïve, central memory and terminally differentiated CD4+ T cell subpopulations in HIV+/cART and HIV-negative subjects

**Fig 1:**
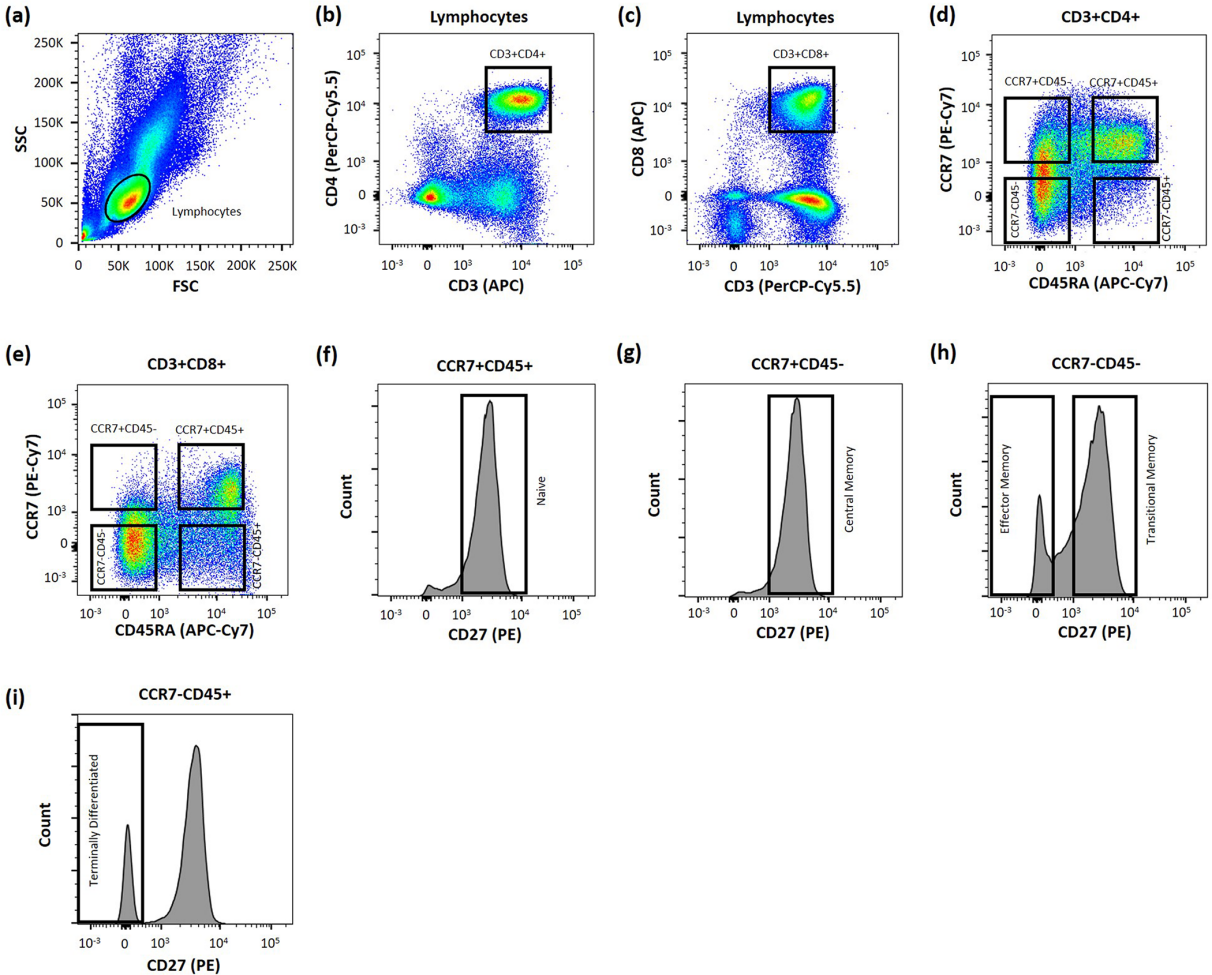
Gating strategy to quantify Glut1-expressing cells within CD4+ and CD8+ T cell subpopulations. (a) Lymphocytes (Circled) were defined using side scatter (SSC) and forward scatter (FSC) characteristics. (b-c) Gating strategy to identify CD3+CD4+ and CD3+CD8+ T cells within the lymphocyte population. (d-e) From the CD4+ and CD8+ T cell population, 4 distinct groups were identified based on CCR7 and CD45RA surface expression. (f-i) These populations were further defined by CCR7, CD45RA and CD27 to identify N (CD27+), CM (CD27+), EM (CD27-), TM (CD27+), and TD populations (CD27-). This gating strategy has been previously described [23,24].

**Fig 2:**
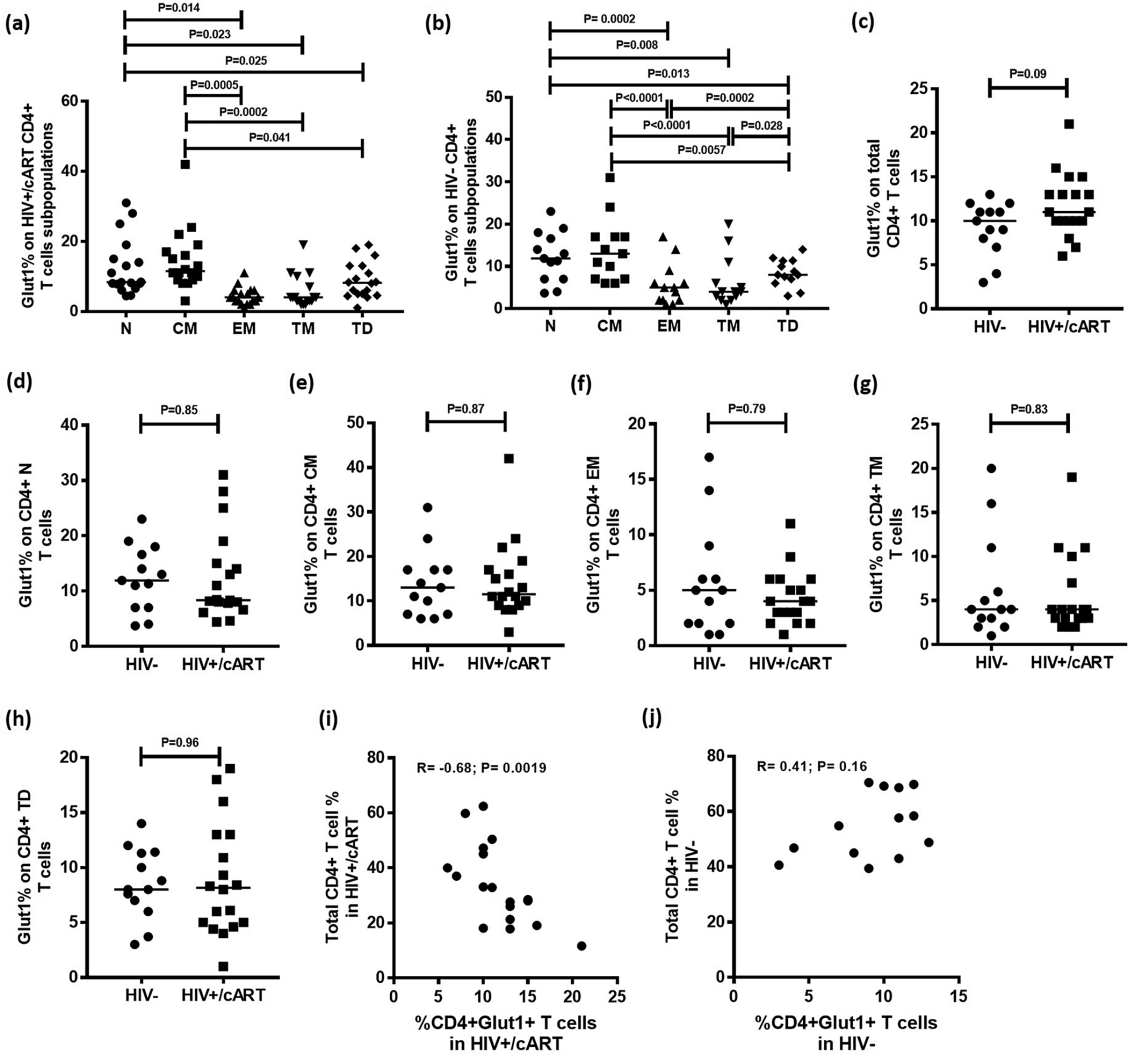
Glut1 expression on total CD4+ T cells and CD4+ subpopulations in HIV-negative and HIV+/cART individuals. (a-b) Median percentage of CD4+Glut1+ T cells within functional CD4+ T cell subpopulations in HIV+/cART and HIV-negative individuals. (c-h) Comparisons of median percentages of CD4+Glut1+ T cells among total and functional CD4+ T cell subpopulations between HIV+/cART and HIV-negative individuals. (i-j) Correlations between percentage of CD4+Glut1+ T cells against total CD4+ T cell percentage among HIV+/cART and HIV-negative individuals.

A conventional gating strategy was used to evaluate Glut1 expression on subpopulations of CD4+ T cells (Fig 1A–1I) according to previously published nomenclature: naïve (N; CCR7+CD45RA+CD27+), central memory (CM; CCR7+CD45RA-CD27+), effector memory (EM; CCR7-CD45RA-CD27-), transitional memory (TM; CCR7-CD45RA-CD27+), and terminally differentiated (TD; CCR7-CD45RA+CD27-) T cells [23]. Among the HIV+/cART participants, Glut1 expression was significantly higher in CM (Median: 11.5%; IQR = 8.5), N (Median: 8.4%; IQR = 8.5) and TD (Median: 8.15; IQR = 8.2) subpopulations when compared with EM (median: 4.0%; IQR = 3.25;) and TM cells (Median: 4.0%; IQR = 4.75;) (Fig 2A). Glut1 expression was not significantly different between CM and N subpopulations (P = 0.5) (Fig 2A). Since elevated cell surface Glut1 is a surrogate marker for glycolytic metabolism, this suggests CD4+ CM, N and TD subpopulations are glycolytically more active than EM and TM subpopulations in HIV+/cART subjects.

To determine if this trend between T cell subpopulations was similar in HIV-negative individuals, identical parameters were measured in a subset of 13 HIV-negative participants. Similar to the HIV+/cART subjects, Glut1 expression was significantly higher in CM (Median: 13.0%; IQR = 10.0), N (Median: 11.9%; IQR = 10.3) and TD (Median: 8.0%; IQR = 4.9) subpopulations when compared with EM (median: 5.0%; IQR = 5.5) and TM (Median: 4.0%; IQR = 6.0) subpopulations. (Fig 2B).

We compared the percentage of Glut1-expressing cells within total CD4+ T cells and CD4+ T cell subpopulations between HIV-negative and HIV+/cART subjects and found no significant differences, although overall there was a trend towards an increased percentage of total CD4+Glut1+ T cells in HIV+/cART subjects (Fig 2C–2H). In HIV+/cART subjects the percentage of CD4+Glut1+ T cells was inversely correlated with the total percentage of CD4+ T cells (R = -0.68; P = 0.0019), but not so for HIV-negative subjects (R = 0.41; P = 0.16) (Fig 2I–2J).

Evaluation of the mean fluorescent intensities (MFI) of Glut1 on CD4+ T cell subpopulations confirmed the above results, illustrating that Glut1 expression is significantly higher among CM (Median: 134.5; IQR = 104.8) and N cells (Median: 138.5; IQR = 74.5) when compared to EM (Median: 104.5; IQR = 60.0) and TM cells (Median: 93.0; IQR = 91.8) among HIV+/cART subjects. Presented as supplementary data (S1 Fig), similar trends existed within blood from HIV-negative individuals, and when comparing across the two groups.

### The levels of Glut1 are highest among naïve and central memory CD8+ T cell subpopulations in HIV+/cART and HIV-negative subjects

**Fig 3:**
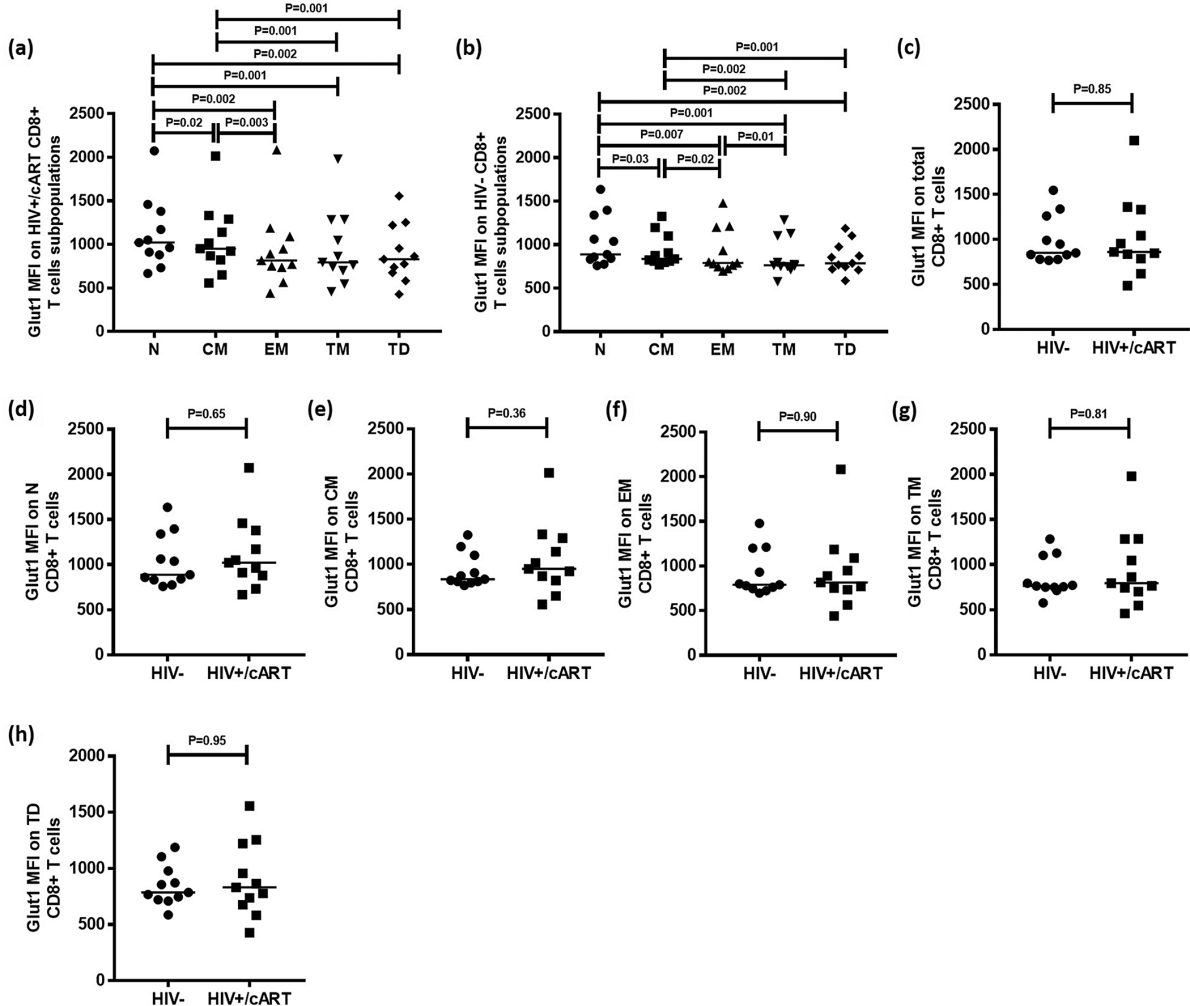
Glut1 expression on total CD8+ T cells and CD8+ T cell subpopulations in HIV-negative and HIV+/cART individuals. (a-b) Median Glut1 MFI of functional CD8+ T cell subpopulations in HIV+/cART and HIV-negative individuals. (c-h) Comparisons of median Glut1 MFI among total and functional CD8+ T cell subpopulations between HIV+/cART and HIV-negative individuals.

A gating strategy designed to identify CD8+ T cell subpopulations was adopted from Chomont et al and Yap et al to evaluate Glut1 among these cell types (Fig 1) [23,24]. Among 11 HIV+/cART participants, Glut1 expression was significantly higher in N (Median: 1022; IQR = 501) and CM (Median: 952; IQR = 469) subpopulations when compared with EM (median: 815; IQR = 354), TM (Median: 794; IQR = 583) and TD subpopulations (Median: 831; IQR = 545) (Fig 3A). Glut1 expression was also significantly lower among the CD8+ CM population when compared to the N population (P = 0.02) (Fig 3A). This suggests that CD8+ CM and N subpopulations are glycolytically more active than EM, TM and TD subpopulations in HIV+/cART subjects.

Identical parameters were measured in a subset of 11 HIV-negative participants. As in HIV+/cART samples, Glut1 expression was significantly higher in N (Median: 889; IQR = 508) than in CM (Median: 837; IQR = 289) subpopulations (P = 0.03), with both these populations having elevated Glut1 MFI in comparison to EM (median: 790; IQR = 455), TM (Median: 763; IQR = 355) and TD subpopulations (Median: 786; IQR = 257) (Fig 3B). Additionally, Glut1 MFI was higher in EM cells than in TM cells (P = 0.01) (Fig 3B).

Glut1 MFI in total CD8+ T cells among HIV-negative (Median: 851; IQR = 482) and HIV+/cART (Median: 861; IQR = 546) was not significantly different (Fig 3C), nor was the MFI in CD8+ T cell subpopulations significantly different between HIV-negative and HIV+/cART groups (Fig 3D–3H).

### Central memory CD4+ T cell subpopulation has the highest mitochondrial density

**Fig 4:**
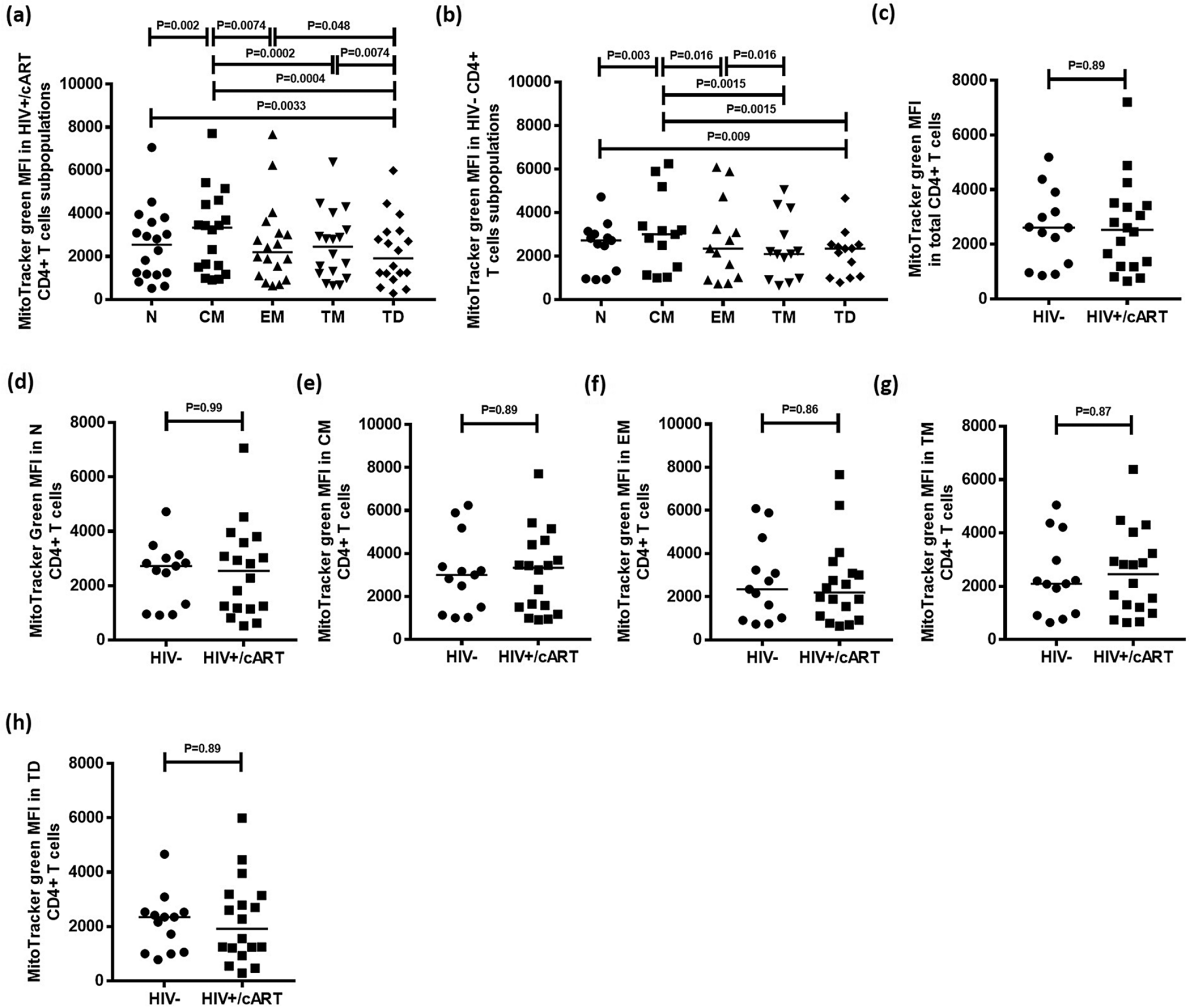
Mitochondrial density measured by MitoTracker green MFI in CD4+ T cells and CD4+ T cell subpopulations in HIV+/cART and HIV-negative individuals. (a,b) Median MitoTracker green MFI in CD4+ T cell subpopulations in HIV+/cART and HIV-negative individuals. (c-h) Comparisons of median MitoTracker green MFI in CD4+ T cells among total and functional CD4+ T cell subpopulations between HIV+/cART and HIV-negative individuals.

MitoTracker green is a mitochondria-selective fluorescent label that accumulates in the mitochondrial matrix and binds to mitochondrial proteins by reacting with free thiol groups of cysteine residues. Using the gating strategy described above, MitoTracker green levels were quantified to determine mitochondrial density in CD4+ T cell subpopulations in HIV+/cART and HIV-negative individuals. Mitochondrial density was highest in CM cells (Median: 3339; IQR = 3042), and lowest in TD cells (Median: 1917; IQR = 2010) (Fig 4A).

To determine whether similar trends existed in HIV-negative individuals, identical parameters were measured in 13 HIV-negative participants. Similar to results obtained from HIV+/cART subjects, mitochondrial density was highest among CM cells (Fig 4B).

Finally, mitochondrial density within total CD4+ T cells was compared between HIV-negative and HIV+/cART individuals. The median MitoTracker green MFI was not significantly different between CD4+ T cells of HIV-negative (Median: 2609; IQR = 2423) and HIV+/cART groups (Median: 2530; IQR = 2256) (P = 0.89) (Fig 4C). There were no significant differences in MitoTracker green MFI between CD4+ T cell subpopulations from HIV-negative and HIV+/cART subjects (Fig 4D–4H).

### Naïve and central memory CD8+ T cell subpopulations have the highest mitochondrial density

**Fig 5:**
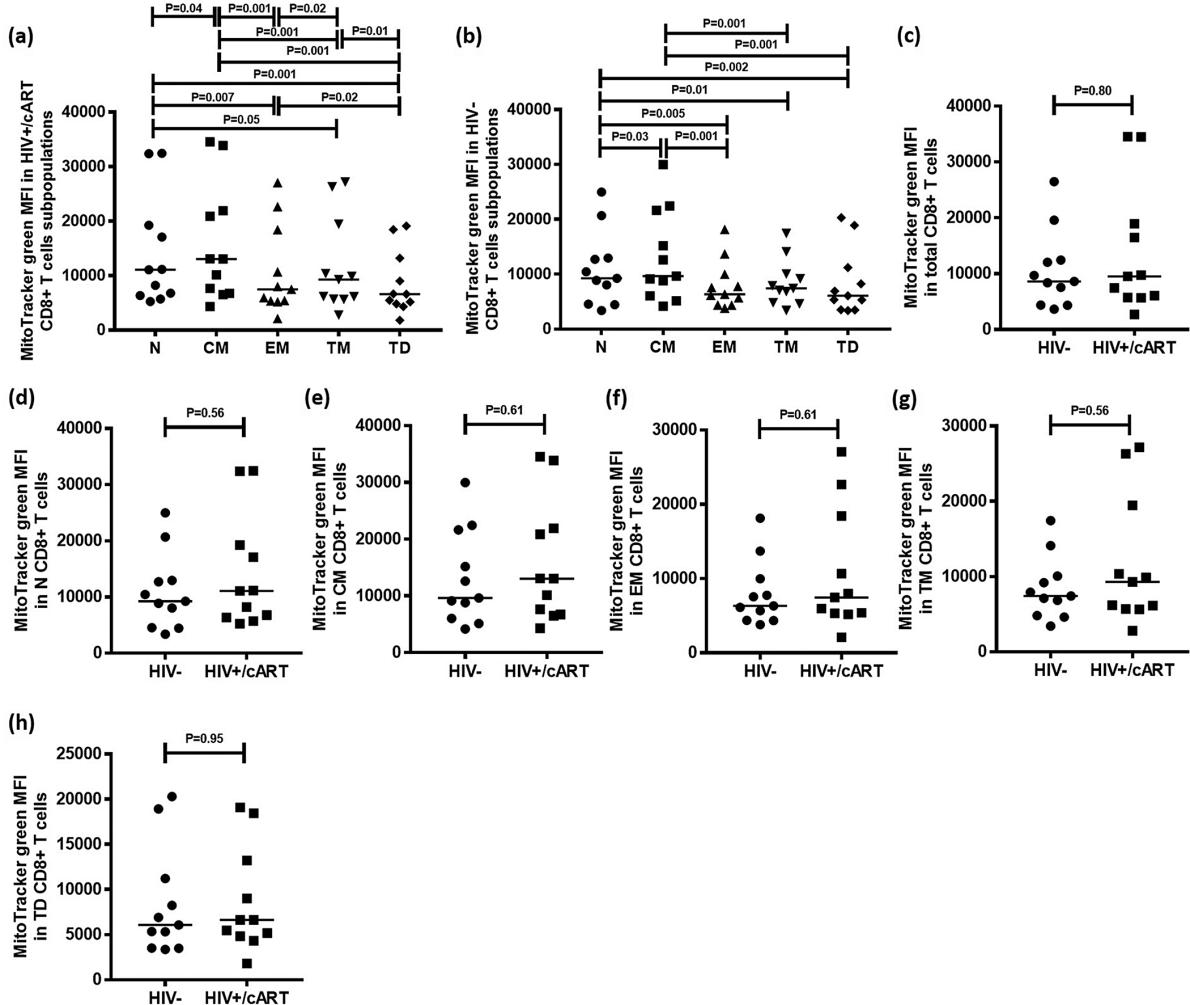
Mitochondrial density measured by MitoTracker green MFI in total CD8+ T cells and CD8+ T cell subpopulations in HIV+/cART and HIV-negative individuals. (a,b) Median MitoTracker green MFI in CD8+ T cell subpopulations in HIV+/cART and HIV-negative individuals. (c-h) Comparisons of median MitoTracker green MFI among total and functional CD8+ T cell subpopulations between HIV+/cART and HIV-negative individuals.

MitoTracker green levels were used to determine mitochondrial density in CD8+ T cell subpopulations in 11 HIV+/cART and HIV-negative individuals. Mitochondrial density was highest in N (Median: 11071; IQR = 12884) and CM (Median: 13042; IQR = 15167) populations, and lowest in EM (Median: 7472; IQR = 13087), TM (Median: 9293; IQR = 13762) and TD subpopulations (Median: 6628; IQR = 8405), with CM cells having significantly higher density than N cells (P = 0.04) (Fig 5A). Mitochondrial density in TD cells was lowest among the subpopulations, with a significant difference when compared to EM (P = 0.02) and TM populations (P = 0.01) (Fig 5A). A significantly higher mitochondrial density was found in TM cells when compared to EM cells (P = 0.02) (Fig 5A).

Among the 11 HIV-negative participants, mitochondrial density was again highest in N (Median: 9259; IQR = 8400) and CM (Median: 9664; IQR = 15621) populations, and lowest in EM (Median: 6337; IQR = 5602), TM (Median: 7402; IQR = 5237) and TD populations (Median: 6067; IQR = 7710) with CM cells having significantly higher mitochondrial density than N cells (P = 0.03) (Fig 5B).

Mitochondrial density MFI within total CD8+ T cells was compared between HIV-negative (Median: 8628; IQR = 8072) and HIV+/cART individuals (Median: 9513; IQR = 13214), with no difference found (P = 0.80) (Fig 3C). As with CD8+ subpopulations, no significant differences in MitoTracker green MFI was found between CD8+ T cell subpopulations from HIV-negative and HIV+/cART subjects (Fig 5D–5H).

### HIV+/cART and HIV-negative subjects have similar mitochondrial membrane potential and oxidative stress levels in their CD4+ and CD8+ T cells

**Fig 6:**
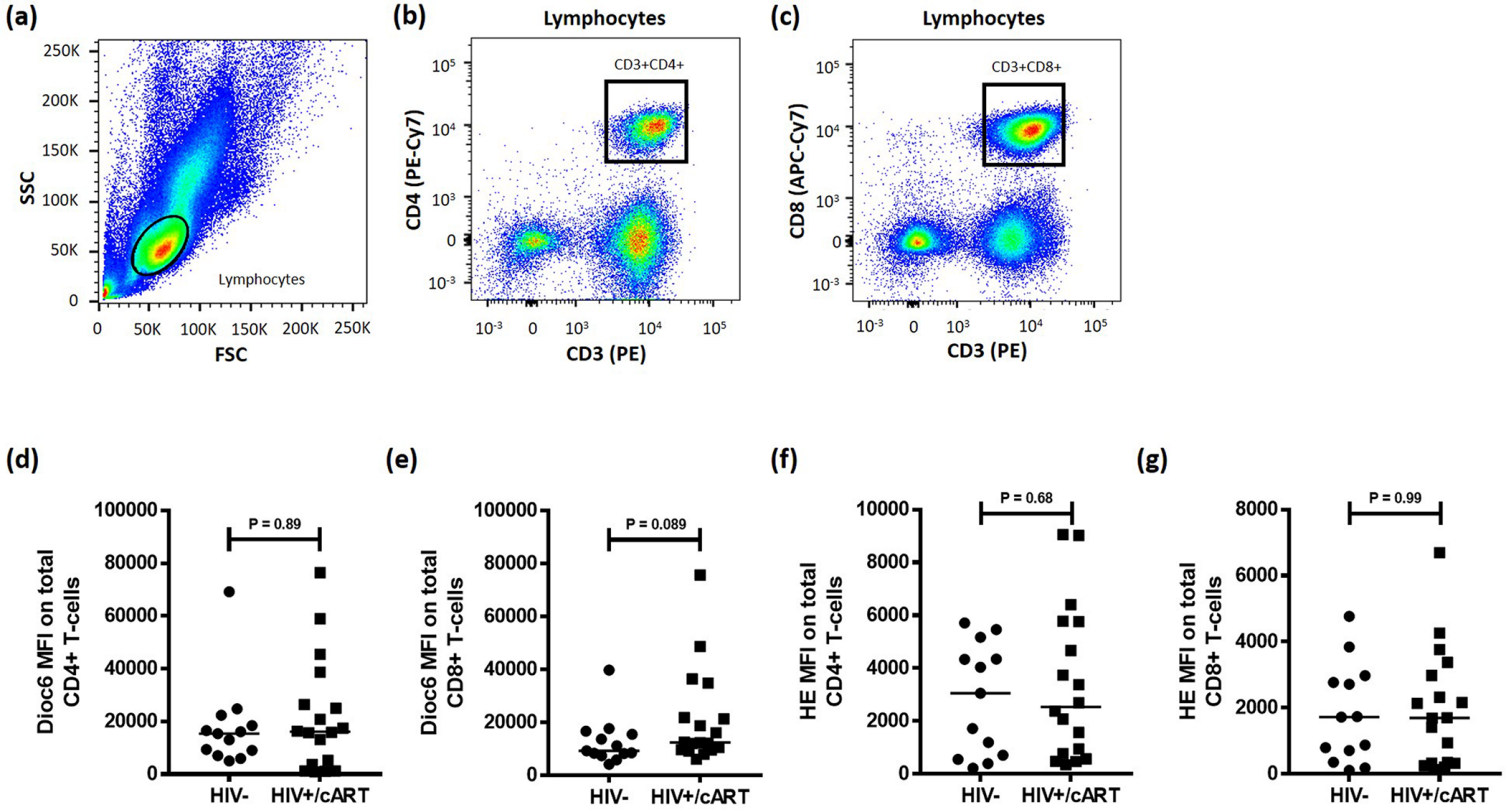
Gating strategy for total CD4+ and CD8+ T cells for analysis of mitochondrial membrane potential in HIV-negative and HIV+/cART individuals. (a) Lymphocytes (Circled) were defined using side scatter (SSC) and forward scatter (FSC) characteristics. (b,c) Gating strategy to identify CD3+CD4+ and CD3+CD8+ T cells within the lymphocyte population using CD3 (PE), CD4 (PE-Cy7), and CD8 (APC-Cy7) antibodies. (d,e) Comparisons of median Dioc_6_ MFI in total CD4+ and CD8+ T cells between HIV+/cART and HIV-negative subjects. (f,g) Comparisons of median HE MFI in total CD4+ and CD8+ T cells between HIV+/cART and HIV-negative subjects.

We evaluated mitochondrial function and oxidative stress in total CD4+ and CD8+ T cells using Dioc_6_ and Hydroethidium (HE) staining. Fig 6A–6C illustrates the gating strategy used to evaluate Dioc_6_ MFI in total CD4+ and CD8+ T cells. Dioc_6_ MFI was similar in HIV-negative (n = 13) and HIV+/cART (n = 18) individuals at 15349 (IQR = 12403) and 16084 (IQR = 26436) respectively in total CD4+ T cell populations (P = 0.89) (Fig 6D). Dioc_6_ MFI was noticeably higher, but not significantly different in CD8+ T cells from HIV+/cART subjects compared to those from HIV-negative individuals (P = 0.089) (Fig 6E).

Median HE MFI was insignificantly different when comparing CD4+ T cells from HIV-negative (Median: 3054; IQR: 4127) and HIV+/cART participants (Median: 2539; IQR: 5048) (P = 0.68) (Fig 6F). The difference in median HE MFI in CD8+ T cells from HIV-negative (Median: 1722; IQR: 2339) and HIV+/cART (Median: 1698; IQR: 2755) individuals was also insignificant (P = 0.99) (Fig 6G). In summary, ROS production is higher in CD4+ T cells than in CD8+ T cells, despite no difference when comparing these cell populations between HIV-negative and HIV+/cART participants.

### Glut1 expression is positively correlated with mitochondrial density and membrane potential in CD4+ and CD8+ T cells

**Fig 7:**
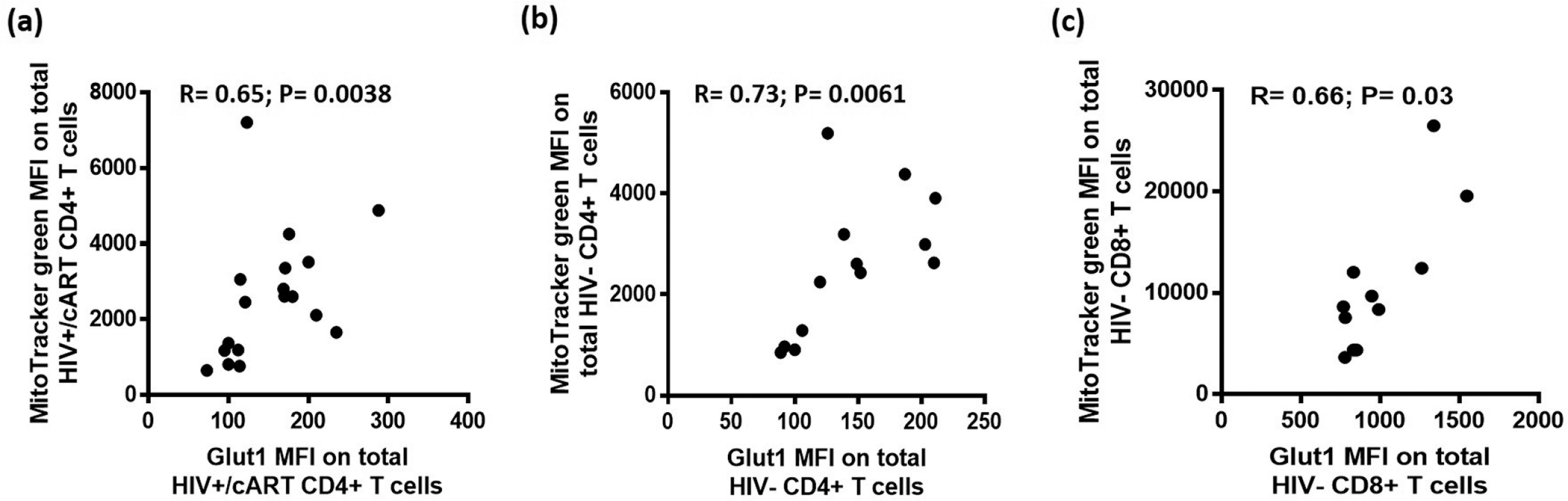
Correlations of Glut1 MFI against the MFI of MitoTracker green. (a-b) Correlations of Glut1 MFI against the MFI of MitoTracker green in CD4+ T cells among HIV+/cART and HIV-negative individuals. (c) Correlation of Glut1 MFI against the MFI of MitoTracker green in CD8+ T cells among HIV-negative individuals.

Significant correlations existed between Glut1 MFI and MitoTracker green MFI (R = 0.65; P = 0.0038) in the 18 HIV+/cART subjects (Fig 7A). A significant correlation was also found between these parameters in the 13 HIV-negative individuals (R = 0.73; P = 0.0061) (Fig 7B). These data suggest that mitochondrial density increases as Glut1 expression increases in CD4+ T cells.

Significant correlations existed between Glut1 MFI and MitoTracker green MFI (R = 0.66; P = 0.03) in CD8+ T cells among the 11 HIV-negative subjects (Fig 7C). Positive correlation was also found between these parameters in 11 HIV+/cART individuals although this was not found to be statistically significant (R = 0.56; P = 0.08).

### Mitochondrial membrane potential is positively correlated with mitochondrial density and reactive oxygen species production

**Fig 8:**
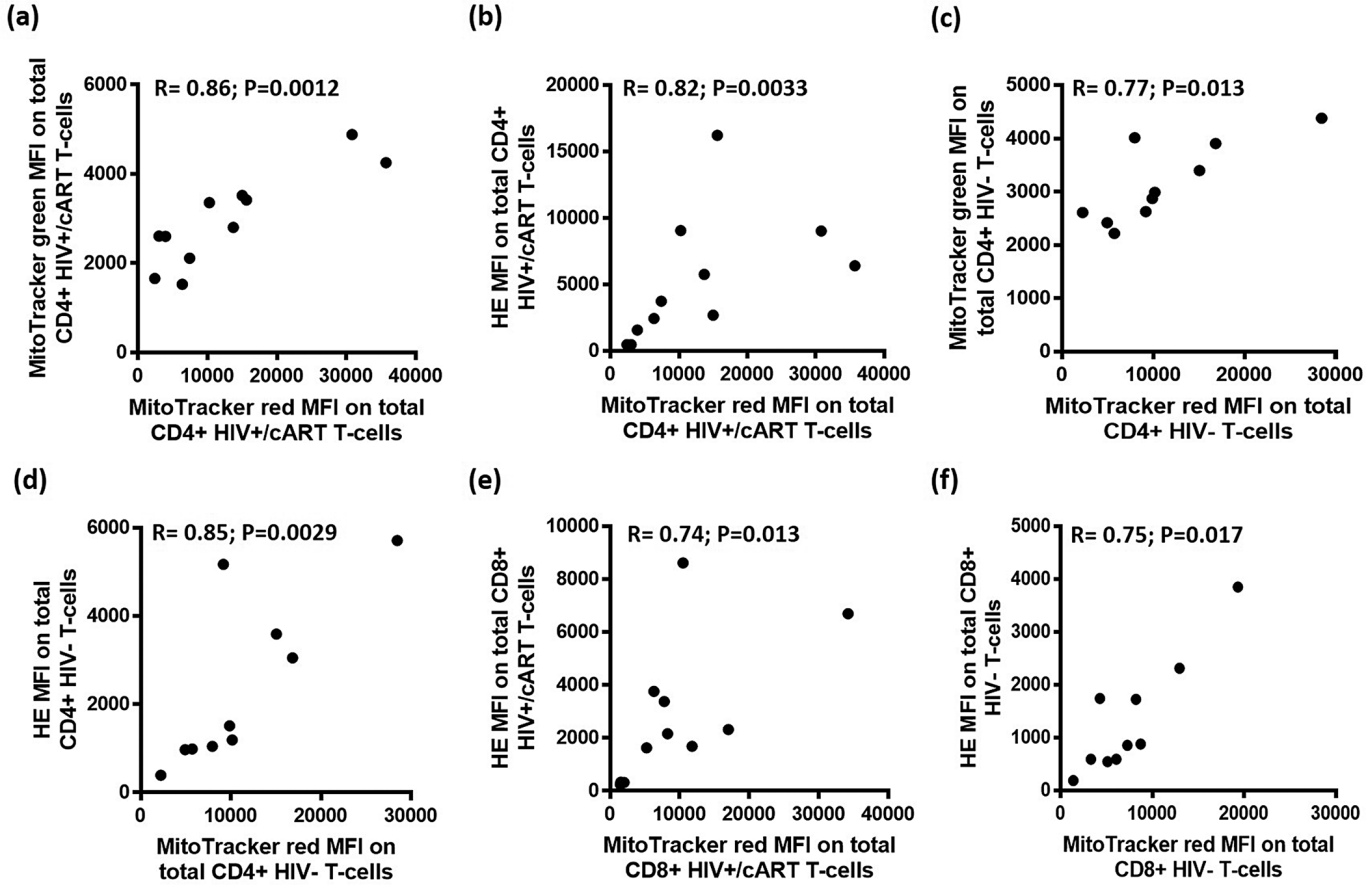
Correlations of MitoTracker red MFI against MitoTracker green and HE MFI. (a,b) The correlation of MitoTracker red MFI against the MFI of MitoTracker green and HE in total CD4+ T cells among HIV+/cART individuals. (c,d) The correlation of MitoTracker red MFI against the MFI of MitoTracker green and HE in total CD4+ T cells among HIV-negative individuals. (e,f) The correlation of MitoTracker red MFI against the MFI of HE in total CD8+ T cells among HIV+/cART and HIV-negative individuals.

MitoTracker red was also used to assess MMP as a mitochondrial specific probe, unlike Dioc6 which stains hyperpolarised membranes of the mitochondria as well as the endoplasmic reticulum and other plasmatic membranes [26,27]. Correlations were performed against mitochondrial mass and oxidative stress in CD4+ T cells. Among the 11 HIV+/cART individuals, significant correlations were found between MMP and both mitochondrial mass (R = 0.86; P = 0.0012), and oxidative stress (R = 0.82; P = 0.0033) (Fig 8A and 8B). Significant correlations were also found between MMP and both mitochondrial mass (R = 0.77; P = 0.013), and oxidative stress (R = 0.85; P = 0.0029) of HIV-negative individuals (Fig 8C and 8D).

Correlations between MMP in CD8+ T cells were also performed against MFI results obtained from mitochondrial mass and oxidative stress analysis. Among the HIV+/cART, significant correlations were found between MMP and oxidative stress in CD8+ T cells (R = 0.74; P = 0.013) (Fig 8E). Significant correlations were found between MMP and HE (R = 0.75; P = 0.017) in the 10 HIV-negative individuals (Fig 8F). This suggests a positive interplay between MMP, mitochondrial density and ROS production in CD4+ and CD8+ T cells.

## Discussion

We report that glycolytic metabolism in virologically suppressed HIV+/cART subjects, measured by cell surface Glut1, is expressed higher in naïve (N) and central memory (CM) CD4+ and CD8+ T cells than in effector memory (EM) and transitional memory (TM) cells. CM cells had the highest mitochondrial density while the lowest was observed in TD cells among both CD4+ and CD8+ T cell subpopulations. These findings were also found in uninfected individuals. Correlation analysis showed a positive relationship between glycolytic metabolism and mitochondrial density in total CD4+ and CD8+ T cells in HIV+/cART individuals. A similar significant positive correlation existed in CD8+ T cells in only HIV-negative subjects. In addition, we observed a significant relationship between mitochondrial membrane potential and oxidative stress in both CD4+ and CD8+ T cells.

Our previous results showed that Glut1 was expressed on a higher percentage of effector (CD45RA+CD27-) and effector-memory (CD45RA-CD27-) CD4 +T cells when compared to naive (CD45RA+CD27+) and memory (CD45RA-CD27+) CD4+ T cells in both HIV+/cART and HIV-negative participants [6]. Through the introduction of the CCR7 cell surface marker and the new gating strategy to identify more specific CD4+ T cell subpopulations, this study expands upon observations made previously [6]. This allowed distinction of naïve cells (CD45RA+CCR7+CD27+) from an uncharacterized population (CD45RA+CCR7-CD27+), as well as distinguishing central memory (CD45RA-CCR7+CD27+) and transitional memory cells (CD45RA-CCR7-CD27+). Furthermore, this strategy allowed identification of terminal differentiated cells (CD45RA+CCR7-CD27-) [23].

Here we demonstrate that Glut1 expression is significantly higher in naïve (N), central memory (CM), and terminally differentiated (TD) CD4+ T cells when compared to effector memory (EM) and transitional memory (TM) T cells in both HIV-negative and HIV+/cART subjects. Notably, in the current study most of the cART-treated HIV-positive subjects had strong CD4+ T cell recovery, a major distinguishing feature from our previous study in which we showed a significantly higher circulating frequency of CD4+Glut1+ T cells in HIV+/cART when compared to HIV-negative individuals [6]. The marked difference in median CD4+ T cell count between these studies potentially impacts the variability in these results. However, the inconsistencies in results when analysing CD4+ T cell subpopulations between the HIV-negative subjects in both studies may also underscore the need for consistency, and choice of cell surface markers when analysing metabolic parameters in T cell subpopulations. Notably for immunometabolic analysis, it is important to consider whether a marker is prone to metabolic regulation. Indeed, CCR7 a chemokine receptor that regulates lymphocyte trafficking is controlled by the phosphatidylinositol-3-OH kinase and mTOR which are evolutionarily conserved regulators of Glut1 and T cell metabolism [28,29].

The general consensus is that effector T cells are generally more glycolytic [12,30,31] while memory and naïve T cells rely on oxidative phosphorylation and fatty acid metabolism [32,33]. However, studies have also demonstrated that memory T cells exhibit high proportions of glucose influx and Acetyl CoA production as a result of increased TCA cycle activity, while EM T cells exhibit low glycolysis and high oxidative phosphorylation in mouse models [34,35]. Support of the former came from observations that showed morphology of cristae within memory T cells are configured to favour oxidative phosphorylation and fatty acid oxidation, while cristae fission in effector T cells leads to reduced oxidative metabolism and increased aerobic glycolysis [36]. While limited data are available that suggest TD CD4+ T cells exhibit heightened Glut1 in comparison to EM cells, high glycolysis in CD8+ T cells has been associated with compromise in the generation of long lived memory cells by driving T cells toward a terminally differentiated state where they quickly develop an apoptotic phenotype in people living with HIV [37,38].

As CM cells constitute a major reservoir site for HIV, and are maintained by homeostatic proliferation [23], it is now speculated that a higher glycolytic propensity may contribute to proliferation of these cells under an inflammatory environment [4]. Previous work has shown that enhanced proliferation is directly correlated with high Glut1 expression in CD4+ T cells [39], supporting a feasible hypothesis that heightened glucose metabolic activities in memory CD4+ T cells might contribute significantly to the maintenance of the HIV reservoir [4,40,41].

Despite generally being considered inactive or “resting”, our data indicate that naive CD4+ T cells may in fact be metabolically active, which might explain why they are also key targets for HIV infection [42,43]. While our current study showed no significant differences in Glut1 expression on CD4+ T cell subpopulations between HIV-negative and HIV+/cART participants, our previous work showed that the percentage of CD4+ naive and memory cells expressing Glut1 was significantly elevated in HIV+/cART patients when compared to HIV-negative participants [6]. However, the current study utilized subjects who had better immune recovery, and further, a different nomenclature employing CCR7 was used to define these populations [6].

We observed no significant differences in Glut1 levels or mitochondrial density in total or subpopulations of CD8+ T cells obtained from HIV+/cART individuals of HIV-negative controls. This is potentially underscored by the high CD4+ T cell recovery in treated HIV-positive subjects. Interestingly, similarly to CD4+ T cells, N and CM CD8+ T cell subpopulations expressed the highest levels of Glut1 and mitochondrial density. While it has been proposed that a high glycolytic CD8+ T cell state may impede the functional development of memory cells [37], studies have shown that elevated glycolysis may provide energy to support memory development [17]. Furthermore, the increased mitochondrial density in memory CD4+ and CD8+ T cells observed in our study may reflect a bioenergetic advantage critical for their recall capacity to mount a robust immune response against pathogens [44–46]. The importance of metabolic reprogramming in immune cell differentiation and immune response has garnered great interest recently. Thus, more work focussing on immunometabolic changes in immune cells in humans in the context of specific infections will help to clarify the importance of immune cell metabolic remodelling during immune responses to viral infection.

In this work, CM T cells had the highest mitochondrial density while TD populations had the lowest. Previous work has shown memory CD4+ and CD8+ T cells generally maintain greater mitochondrial mass than effector T cells, indicating their dependence on oxidative phosphorylation [33,47,48]. Upon IL-2 and IL-15 stimulation, memory cells enter a quiescent state when unstimulated by disease, becoming dependent on fatty acid β-oxidation, maintaining energy production through oxidative phosphorylation and preventing mitochondrial degradation [31,49]. High mitochondrial content and maintained energy production ensures rapid reactivation upon reinfection [50].

Remarkably we found that terminally differentiated cells have high Glut1 expression, but low mitochondrial mass when compared with other CD4+ subpopulations. Generally, TD cells exhibit an exhausted phenotype in HIV infected people and is associated with CD4+ T cell depletion in the cervix [51]. It is reasonable to hypothesize that a high glycolytic metabolism and low mitochondrial mass in the context of HIV infection shifts cellular metabolism to a lower energy producing pathway in favour of biosynthetic precursors required for cellular proliferation. This may consequently result in metabolic exhaustion and ultimately cell death [3]. Spare respiratory capacity, defined as the extra mitochondrial capacity available in a cell used to produce energy under conditions of increased work or stress, is generally highest in memory T cells and is thought to be important for long term cellular survival and function [50]. Our data suggest that a lower mitochondrial density lowers the spare respiratory capacity of the TD cell, thereby reducing its survivability.

When we compared mitochondrial mass, mitochondrial membrane potential and ROS in total CD4+ and CD8+ T cells between HIV+/cART and HIV-negative individuals, we observed no significant differences. This does not corroborate the results from Yu and colleagues [21], who demonstrated that ROS and mitochondrial mass are significantly increased in HIV+/cART participants. Several explanations may explain this discrepancy. Firstly, Yu et al [21], used JC-1 (5,5,6,6tetrachloro-1,1,3,3-tetraethylbenzimidazolo carbocyanine iodide), a polychromatic fluorescent probe to measure MMP, and DCFH (2, 7dichlorodihydrofluorescein), which measures total ROS, unlike HE which detects superoxide species. Furthermore, in their study participants were only receiving cART for a short time (0.5 to 3 years), while in our study subjects were virologically suppressed for more than 6 years [21]. Furthermore, we cannot exclude the impact of different cART regimens between these studies and the degree of CD4+ T cell recovery. These discrepancies highlight the importance of normalization of methodologies to assess mitochondrial functions and biogenesis of immune cells in disease settings, as well as to carefully examine the patient groups enrolled and clinical factors that may affect specific metabolic parameters.

One limitation of this study is the modest sample size. In addition, the generally wide variance across the T cell subpopulations for the markers analysed may limit data interpretation. Furthermore, details of current and past treatment regimens would have been informative because although the direct impact of cART on metabolism in immune cells is largely unknown, earlier cART can influence mitochondrial metabolism in insulin sensitive tissues [52,53]. Furthermore, fasting state, supplement intakes like resveratrol, and medication like metformin can regulate mTOR [54–57]. These are important considerations that should be kept in mind in for subsequent studies and will help to improve our understanding on how metabolism in immune cells dictates their functions and contributes to the outcome of HIV infection.

In conclusion, we found distinct Glut1 cell surface levels and mitochondrial mass in specific CD4+ and CD8+ T cell subpopulations in HIV-negative and HIV+/cART individuals. Importantly, central memory CD4+ and CD8+ T cells in HIV+/cART subjects are characterized by high Glut1 expression and high mitochondrial mass. This is a characteristic feature of highly metabolically active cells, capable of responding rapidly to antigenic rechallenge. We observed no significant differences in mitochondrial dynamics in total CD4+ and CD8+ T cells or their subpopulations between HIV-negative and HIV+/cART subjects, which may be explained by the high immune recovery of our treated HIV-positive group. This work along with providing novel insights into metabolic features of T cells in HIV-positive and HIV-negative persons will provide valuable tools to investigate novel mechanisms associated with immune responses to HIV infection.

## Supporting information

S1 Fig Glut1 MFI on total CD4+ T cells and CD4+ subpopulations in HIV-negative and HIV+/cART individuals. (a-b) Median Glut1 MFI on CD4+ T cell subpopulations in HIV-negative and HIV+/cART subjects. (c-h) Comparisons of median Glut1 MFI among total CD4+ populations and CD4+ subpopulations between HIV+/cART and HIV-negative subjects.
(TIF)
