# Supplementary figures and images for: Assessment of metabolic and mitochondrial dynamics in CD4+ and CD8+ T cells in virologically suppressed HIV-positive individuals on combination antiretroviral therapy
Source: PLoS One. 2017 Aug 30;12(8):e0183931. doi: 10.1371/journal.pone.0183931 (PMC5576743; doi:10.1371/journal.pone.0183931)

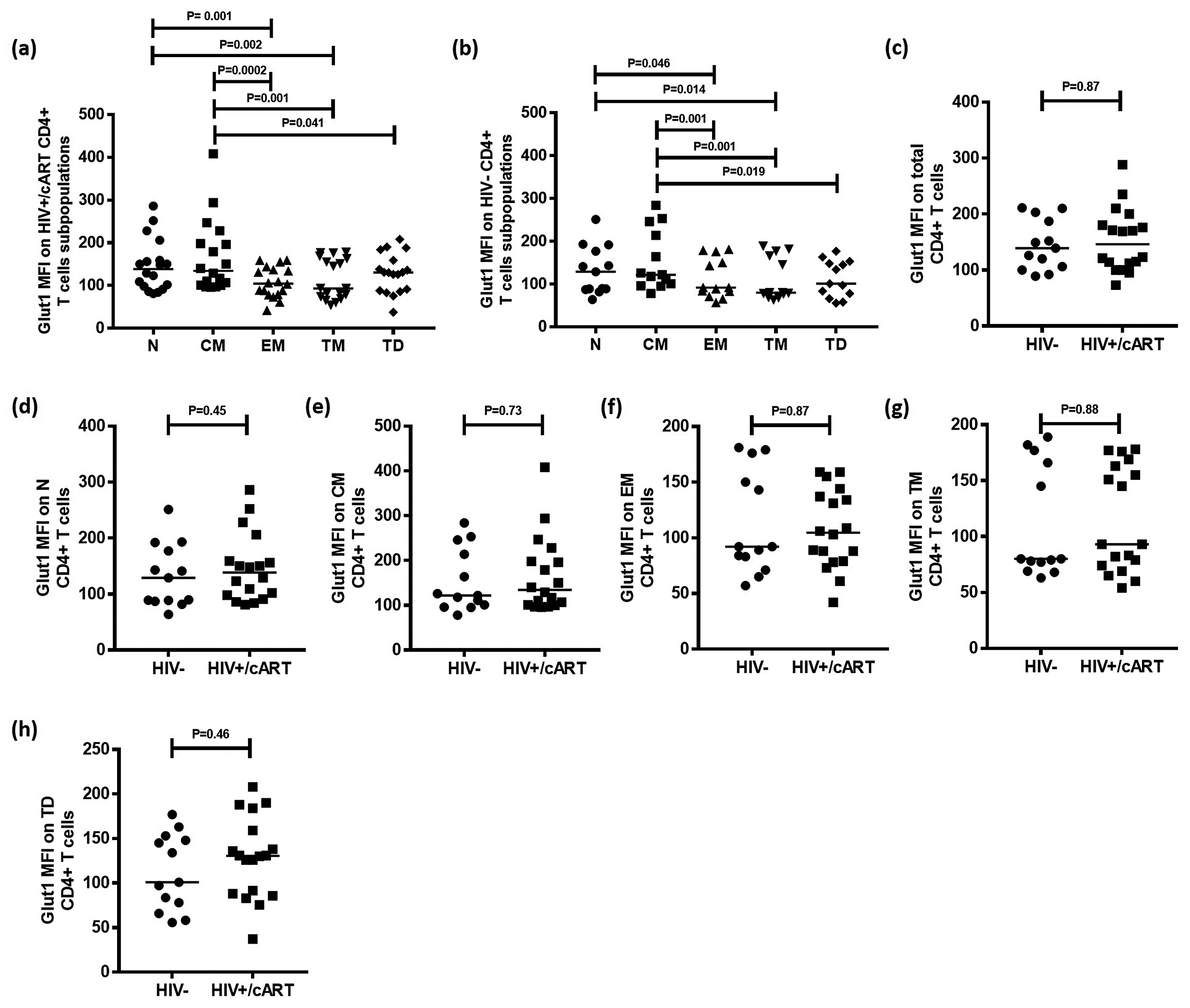

Supplement: S1 Fig — (a-b) Median Glut1 MFI on CD4+ T cell subpopulations in HIV-negative and HIV+/cART subjects. (c-h) Comparisons of median Glut1 MFI among total CD4+ populations and CD4+ subpopulations between HIV+/cART and HIV-negative subjects. (TIF) [file pone.0183931.s001.tif]
